# Risk of Hepatitis B Reactivation Following Corticosteroids for Tuberculous Meningitis

**DOI:** 10.1093/ofid/ofag575

**Published:** 2026-09-02

**Authors:** Joseph Donovan, Nguyen Thi Anh Thu, Ho Dang Trung Nghia, Nguyen Duc Bang, Dong Huu Khanh Trinh, Lam Hong Bao Ngoc, Pham Kieu Nguyet Oanh, Evelyne Kestelyn, Tran Thai Hung, Ronald B Geskus, Nguyen Thuy Thuong Thuong, Nguyen Hoan Phu, Guy E Thwaites

**Affiliations:** Oxford University Clinical Research Unit, Hospital for Tropical Diseases, Ho Chi Minh City, Vietnam; Centre for Tropical Medicine and Global Health, Nuffield Department of Medicine, University of Oxford, Oxford, UK; Oxford University Clinical Research Unit, Hospital for Tropical Diseases, Ho Chi Minh City, Vietnam; Hospital for Tropical Diseases, Ho Chi Minh City, Vietnam; Pham Ngoc Thach University of Medicine, Ho Chi Minh City, Vietnam; Pham Ngoc Thach Hospital for Tuberculosis and Lung Disease, Ho Chi Minh City, Vietnam; Oxford University Clinical Research Unit, Hospital for Tropical Diseases, Ho Chi Minh City, Vietnam; Department of Biomedical Engineering, King's College London, London, UK; Oxford University Clinical Research Unit, Hospital for Tropical Diseases, Ho Chi Minh City, Vietnam; Hospital for Tropical Diseases, Ho Chi Minh City, Vietnam; Oxford University Clinical Research Unit, Hospital for Tropical Diseases, Ho Chi Minh City, Vietnam; Centre for Tropical Medicine and Global Health, Nuffield Department of Medicine, University of Oxford, Oxford, UK; Oxford University Clinical Research Unit, Hospital for Tropical Diseases, Ho Chi Minh City, Vietnam; Oxford University Clinical Research Unit, Hospital for Tropical Diseases, Ho Chi Minh City, Vietnam; Centre for Tropical Medicine and Global Health, Nuffield Department of Medicine, University of Oxford, Oxford, UK; Oxford University Clinical Research Unit, Hospital for Tropical Diseases, Ho Chi Minh City, Vietnam; Centre for Tropical Medicine and Global Health, Nuffield Department of Medicine, University of Oxford, Oxford, UK; Oxford University Clinical Research Unit, Hospital for Tropical Diseases, Ho Chi Minh City, Vietnam; Centre for Tropical Medicine and Global Health, Nuffield Department of Medicine, University of Oxford, Oxford, UK; School of Medicine, Vietnam National University of Ho Chi Minh City, Ho Chi Minh City, Vietnam; Oxford University Clinical Research Unit, Hospital for Tropical Diseases, Ho Chi Minh City, Vietnam; Centre for Tropical Medicine and Global Health, Nuffield Department of Medicine, University of Oxford, Oxford, UK

**Keywords:** corticosteroids, hepatitis B, liver, reactivation, tuberculous meningitis

## Abstract

High-dose corticosteroids may cause reactivation or relapse of hepatitis B virus (HBV) infection. We investigated the virologic and clinical consequences of 6–8 weeks dexamethasone in HBV infected/exposed/unexposed HIV-1 negative adults with tuberculous meningitis enrolled into a placebo-controlled trial. Dexamethasone was not associated with viral reactivation or increased liver injury.

Corticosteroids are used to treat a wide variety of inflammatory conditions, including those caused by infectious diseases. For example, short courses of high-dose corticosteroids, given intravenously or orally, have been shown to improve outcomes in children and adults with tuberculous meningitis. However, the immune suppression induced by corticosteroids may result in the reactivation of latent coinfections, complicating management of the primary infection.

Globally, >300 million individuals have chronic hepatitis B virus (HBV) infection [1], and 2 billion have resolved HBV infection [2]; Southeast Asia is disproportionately affected. Approximately 10.5% of Vietnam's adult population is with HBV [3]. Individuals receiving corticosteroids for tuberculous meningitis and other conditions are therefore often with HBV.

The risks of HBV reactivation or relapse as a result of short courses of high-dose corticosteroids are not well defined. In a retrospective study in South Korea of 198 patients with chronic hepatitis B and asthma or COPD, HBV reactivation (defined as an absolute or relative alanine transaminase [ALT] increase, and an absolute HBV increase) developed in 8 of 72 (11.1%) individuals who received continuous or intermittent systemic corticosteroids versus 4 of 126 (3.2%) individuals who received inhaled corticosteroids [4]. In 220 adults with rheumatic disease, 3/101 (3.0%) receiving corticosteroids alone (corticosteroid duration 22–30 months) and 3/119 (2.5%) receiving corticosteroids with additional immunosuppression, converted to HBV-DNA-positive [5]. However, a retrospective study of 258 patients with resolved HBV who received corticosteroids (prednisone 20–60 mg/day) plus additional immunosuppression (in 192 [74.4%] cases), no individuals developed hepatitis flare or became HBV-DNA-positive [6].

Corticosteroid dose and duration, and concomitant use of additional immunosuppressants, represent important considerations when considering HBV reactivation risk. Clinical guidelines for prevention and treatment of HBV reactivation highlight that in individuals with previous HBV (HBsAg negative, anti-HBc positive), corticosteroid therapy for ≥4 weeks that is moderate dose (10–20 mg prednisone daily or equivalent) or high dose (>20 mg prednisone daily or equivalent), indicates a 1–10% risk of reactivation [7].

To investigate whether the corticosteroid regimen recommended globally for tuberculous meningitis treatment increases the risks for HBV reactivation or relapse, we performed a longitudinal evaluation of liver function tests (LFTs), HBV viral load, and other outcomes in Vietnamese adults with tuberculous meningitis randomized to corticosteroids or placebo in a clinical trial.

## METHODS

HIV-negative adults (≥18 years) with suspected tuberculous meningitis were enrolled into a leukotriene A4 hydrolase (LTA4H) genotype-stratified randomized, placebo-controlled trial of corticosteroids (LAST ACT trial; ClinicalTrials.gov NCT03100786). Detailed methods are provided in the published protocol [8], and the report of the primary results [9]. Written informed consent to enter the LAST ACT trial was obtained from all participants (or from a relative if they were incapacitated). Approvals for the LAST ACT trial were obtained from local and national ethics and regulatory authorities in Vietnam, and from the Oxford Tropical Research Ethics Committee in the UK.

All participants underwent rapid LTA4H genotyping after enrollment [8]: CC/CT-genotype participants (87% of the study cohort) were randomized to dexamethasone or placebo; TT-genotype participants received open-label dexamethasone. Participants with MRC grade II or III (moderate to severe) disease received intravenous dexamethasone/placebo for 4 weeks (0.4 mg/kg/day first week, 0.3 mg/kg/day second week, 0.2 mg/kg/day third week, and 0.1 mg/kg/day fourth week) and then oral administration for 4 weeks, starting at 4 mg/day and decreasing by 1 mg each week. Participants with grade I (mild) disease received intravenous administration for 3 weeks (0.3 mg/kg/day first week, 0.2 mg/kg/day second week, and 0.1 mg/kg/day third week) and then oral administration for 3 weeks, starting at 3 mg per day and decreasing by 1 mg each week. All trial participants also received 12 months of oral antituberculosis chemotherapy following Vietnamese national guidelines (2 months rifampicin 10 mg/kg/day, isoniazid 5 mg/kg/day, pyrazinamide 2 g/day, ethambutol 20 mg/kg/day; 10 months daily rifampicin, isoniazid, ethambutol, at the same doses).

All trial participants were tested for hepatitis B (HBsAg, HBcAb) and hepatitis C (core antibody [cAb]) at baseline. LFTs were performed at least weekly until discharge and then 3-monthly until 12 months from randomization. HBsAg or HBcAb positive participants also had HBV viral load quantification (real-time PCR; Cobas E4800/6800, Roche, USA) at enrollment, repeated during and/or after receiving dexamethasone. HBV viral loads, measured 14–104 days after enrollment, were analyzed, to maximally capture individuals undergoing repeat HBV load measurement during, or after, receiving adjunctive dexamethasone.

Baseline HBsAg positive, CC/CT-genotype participants were unblinded; those allocated dexamethasone were treated with tenofovir 300 mg daily until at least 12 months after completion of dexamethasone, as were all TT-genotype HBsAg positive participants who all received dexamethasone (Supplementary Figure 1). All other participants received blinded dexamethasone/placebo without tenofovir. We categorized participants into 3 groups:

1. Chronic/active HBV infection: HBsAg positive;
2. Resolved HBV infection: HBsAg negative, HBcAb positive;
3. Unexposed: HBcAb negative.

Baseline data, longitudinal laboratory/virologic data, and clinical endpoints were compared across these groups. Liver injury was defined as elevation of blood ALT ≥3 times upper limit of normal (ULN) with symptoms, or ≥5 times ULN, or bilirubin >34 µmol/L without symptoms (following the definition of drug-injury liver injury used in the LAST ACT trial). No sample size calculation was performed for this exploratory substudy. Data were analyzed using R (version 4.4.2; R Project for Statistical Computing) [10].

## RESULTS

**Figure 1.**
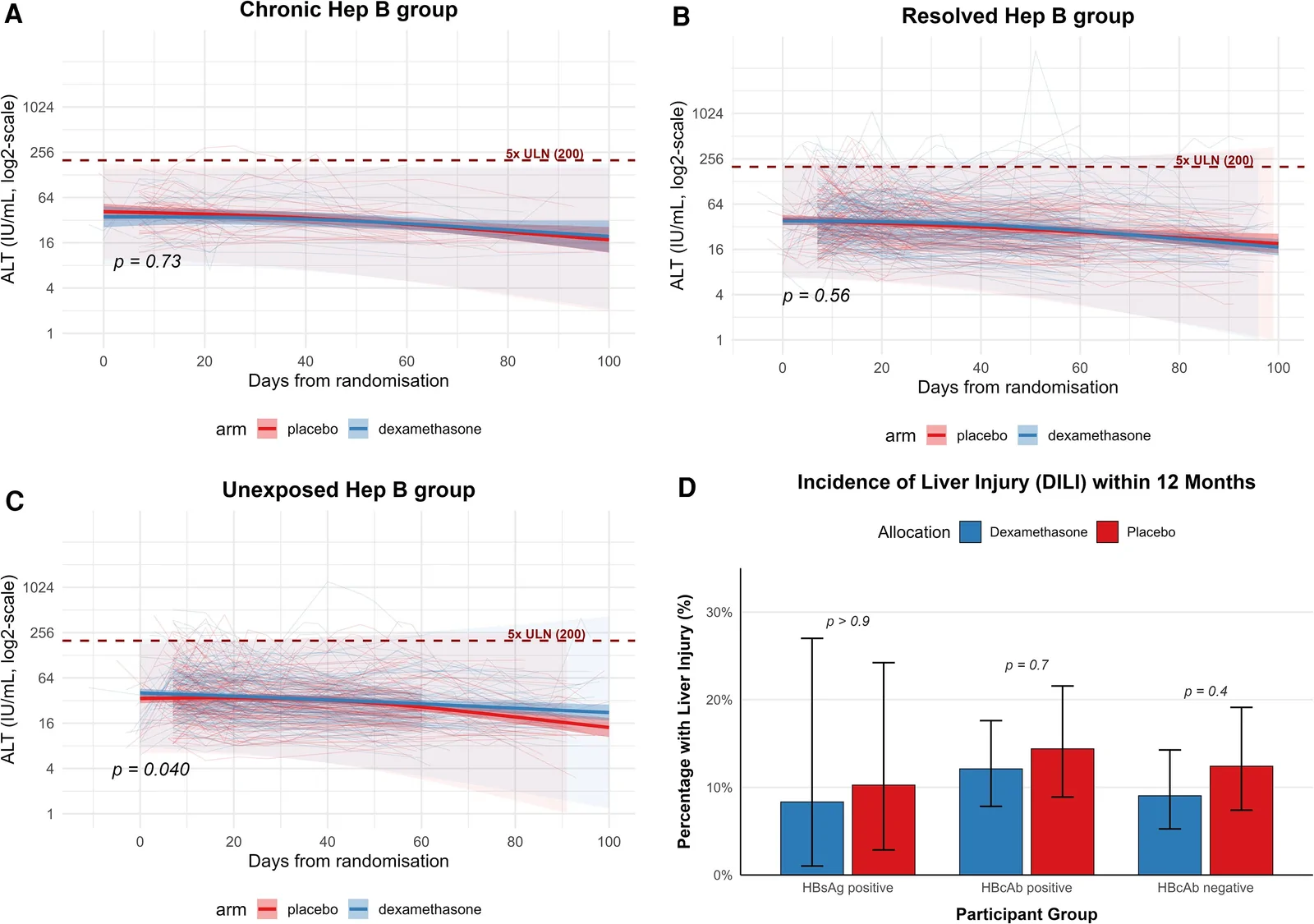
ALT rise and liver injury; ALT trend over 100 d by study arm, for (*A*) HBsAg positive, (*B*) HBsAg negative/HBcAb positive, (*C*) HBcAb negative groups, and (*D*) percentage of participants with liver injury by study arm. *A*, Chronic Hep B group. *B*, Resolved Hep B group. *C*, Unexposed Hep B group. *D*, Percentage of participants with liver injury by study arm. The ALT threshold of 200 IU/L, shown on (*A-C*) as a horizontal dashed line, was exceeded by 2 participants in the chronic hepatitis B group (0 allocated to dexamethasone, 2 allocated to placebo), 35 participants in the resolved hepatitis B group (22 allocated to dexamethasone, 13 allocated to placebo), and 26 participants in the unexposed hepatitis B group (15 allocated to dexamethasone, 11 allocated to placebo). For panels (*A*), (*B*), and (*C*), a linear-mixed effects model was fitted for log-transformed ALT values that included time (days since randomization) as a continuous variable modeled using a natural cubic spline. The vertical dashed line indicates day 60. The thick central solid lines show model-estimated population mean trajectories derived from the linear-mixed effects model. The inner darker shaded bands represent the 95% confidence intervals around the population mean. The lighter outer shaded bands represent the 95% prediction intervals, reflecting expected variation for individual participant trajectories. The faint background lines represent individual observed longitudinal trajectories for each patient. The horizontal dashed line represents an ALT of 200 IU/L. *P* values are from a likelihood ratio test comparing models with and without a treatment-by-time interaction. For (*D*), liver injury was defined as elevation of blood ALT ≥3 times ULN with symptoms, or ≥5 times ULN, or bilirubin >34 µmol/L without symptoms. Colored bars show percentage of participants in that group, and black lines show 95% confidence intervals. Abbreviations: ALT, alanine transaminase; ULN, upper limit of normal.

From February 12, 2018 to March 9, 2023, baseline HBV tests were conducted in 699/702 enrolled trial participants. Sixty-three (9.0%) were HBsAg positive; 322 (46.1%) were HBcAb positive/HBsAg negative; and 314 (44.9%) were HBcAb negative. Baseline and follow-up clinical and laboratory data are shown by dexamethasone/placebo allocation in Table 1. Kinetics of ALT for HBsAg positive, HBsAg negative/HBcAb positive, and HBcAb negative groups are shown in Figure 1. Fourteen participants had hepatitis C antibodies detected (indicating hepatitis C exposure); none of these participants were HBsAg positive. HBsAg positive participants tended to be older than HBcAb or HBV unexposed participants; tuberculous meningitis severity was similar between the groups.

**Table 1.** Hepatitis B Testing at Baseline and During Corticosteroid Therapy

	HBsAg+ (chronic/active HBV)				HBsAg−, HBcAb+ (resolved HBV)				HBcAb− (unexposed HBV)			
	Dexamethasone N = 24		Placebo N = 39		Dexamethasone N = 190		Placebo N = 132		Dexamethasone N = 177		Placebo N = 137	
	N	Statistic (IQR, %)	N	Statistic (IQR, %)	N	Statistic (IQR, %)	N	Statistic (IQR, %)	N	Statistic (IQR, %)	N	Statistic (IQR, %)
Baseline (precorticosteroids)												
Age (years)	24	47 (39, 65)	39	45 (35, 59)	190	52 (39, 62)	132	47 (38, 59)	177	40 (28, 55)	137	41 (29, 58)
Sex (%)	24	…	39	…	190	…	…	…	177	…	137	…
Female	…	9 (37.5%)	…	16 (41.0%)	…	68 (35.8%)	…	45 (34.1%)	…	72 (40.7%)	…	56 (40.9%)
Male	…	15 (62.5%)	…	23 (59.0%)	…	122 (64.2%)	…	87 (65.9%)	…	105 (59.3%)	…	81 (59.1%)
MRC TBM grade (%)	24	…	39	…	190	…	132	…	177	…	137	…
1	…	11 (45.8%)	…	18 (46.2%)	…	82 (43.2%)	…	47 (35.6%)	…	72 (40.7%)	…	67 (48.9%)
2	…	11 (45.8%)	…	17 (43.6%)	…	95 (50.0%)	…	70 (53.3%)	…	84 (47.5%)	…	64 (46.7%)
3	…	2 (8.3%)	…	4 (10.3%)	…	13 (6.8%)	…	15 (11.4%)	…	21 (11.9%)	…	6 (4.4%)
Hepatitis C Ab+ (%)	24	0 (0%)	39	0 (0%)	190	4 (2.1%)	132	4 (3.0%)	177	5 (2.8%)	135	1 (0.7%)
ALT (IU/L)	24	31.5 (20.3, 48.0)	39	31.3 (21.3, 49.2)	190	24.4 (18.0, 44.0)	131	27.2 (18.6, 46.1)	175	26.0 (16.0, 43.1)	137	29.0 (20.0, 44.0)
Bilirubin (μmol/L)	24	16.3 (12.1, 23.0)	39	13.8 (10.7, 20.0)	189	12.6 (8.9, 18.4)	130	14.8 (11.1, 21.5)	175	13.4 (9.9, 18.9)	137	13.7 (9.5, 17.7)
Hepatitis B viral load (%)	21	…	26	…	183	…	125	…	3	…	3	…
Detectable	…	20 (95.2%)	…	23 (88.5%)	…	3 (1.6%)	…	1 (0.8%)	…	0 (0%)	…	0 (0%)
Undetectable	…	1 (4.8%)	…	3 (11.5%)	…	180 (98.4%)	…	124 (99.2%)	…	3 (100%)	…	3 (100%)
Until 3 m postbaseline (postcorticosteroids)												
Peak ALT (IU/L)	24	51.7 (39.9, 84.8)	39	56.0 (41.5, 87.1)	190	64.0 (35.5, 105.0)	132	60.3 (37.3, 103.3)	177	59.0 (38, 104)	137	56.0 (34, 101)
Peak bilirubin (μmol/L)	24	21.8 (16.9, 48.9)	39	18.1 (13.3, 25.1)	190	15.8 (11.8, 22.6)	132	18.1 (12.8, 27.3)	177	15.8 (12.4, 22.8)	137	15.9 (12.5, 21.8)
Repeat HBV by 3 m (%)^a^	11	…	11	…	79	…	50	…	0	…	0	…
Detectable	…	7 (63.6%)	…	11 (100%)	…	2 (2.5%)	…	2 (4.0%)		Not performed		Not performed
Undetectable	…	4 (36.4%)	…	0 (0%)	…	77 (97.5%)	…	48 (96.0%)				
Mortality by 3 m	24	3 (12.5%)	39	4 (10.3%)	190	24 (12.6%)	132	24 (18.2%)	177	26 (14.7%)	137	16 (11.7%)
Until 12 m postbaseline												
Incidence of drug induced liver injury	24	2 (8.3%)	39	4 (10.3%)	190	23 (12.1%)	132	19 (14.4%)	177	16 (9.0%)	137	17 (12.4%)
Mortality by 12 m	24	5 (20.8%)	39	7 (17.9%)	190	39 (20.5%)	132	34 (25.8%)	177	38 (21.5%)	137	25 (18.2%)

ALT, alanine transaminase; HBV, hepatitis B virus; MRC, Medical Research Council; TBM, tuberculous meningitis.

^a^Denominator given as those who had hepatitis B viral load testing performed within the specified time window. A time window of 14–104 d is used for a repeat HBV result. Individuals with chronic/active hepatitis B who were allocated to steroids were treated for hepatitis B with tenofovir following study procedures. Individuals with chronic/active hepatitis B who were allocated to placebo were treated following local guidelines, and would not all receive tenofovir. N = number of participants included in that statistic. Summary statistic = the median (1st and 3rd quartile) value for continuous data, and the number and frequency (%) of participants with the characteristic for categorical data.

HBV was detectable in 43/47 (91.5%) of HBsAg positive participants at baseline, compared with 4/308 (1.3%) of HBcAb participants. Baseline ALT concentrations tended to be higher in the HBsAg participants than the other groups.

Three months after randomization, in the HBsAg positive participants, HBV was detectable in 11/11 (100%) retested in the placebo group and 7/11 (63.6%) tested in the dexamethasone/tenofovir group. Dexamethasone was not associated with increased HBV detection in the HBcAb positive participants. Of the 18 participants in the chronic hepatitis B group who experienced detectable HBV at repeat testing by 3 months, only 10 measurements were >2000 copies/ml (for participants allocated to dexamethasone; 2640, 14 600 and 25 500, and for participants allocated to placebo; 6710, 8310, 23 700, 65 800, 8 970 000, 10 100 000 and 202 000 000, respectively). Of the 4 participants in the resolved hepatitis B group who experienced detectable HBV at repeat testing by 3 months, all measurements were <100 copies/ml.

Supporting the safety of dexamethasone for all participants, there was no suggestion of increased episodes of liver injury within 12 months from randomization in HBsAg positive participants (2 [8.3%] dexamethasone; 4 [10.3%] placebo, χ^2^ test *P* > .9). This compares with HBcAb positive participants (23 [12.1%] dexamethasone; 19 [14.4%] placebo, χ^2^ test *P* = .7), and HBcAb negative (unexposed) participants (16 [9%] dexamethasone; 17 [12.4%] placebo, χ^2^ test *P* = .4). Overall, the incidence of liver injury until 12 months postbaseline was 81/699 (11.6%). Twelve-month mortality was similar across the groups.

## DISCUSSION

Six to eight weeks of high-dose dexamethasone, given with tenofovir in HBsAg positive adults, did not increase HBV reactivation, episodes of liver injury, or mortality in HBV infected/exposed/unexposed adults with tuberculous meningitis. A strength of our study is that it prospectively enrolled a large population and randomized individuals to corticosteroids or placebo. Study participants were carefully characterized, and underwent longitudinal monitoring of liver function and hepatitis B viral load, and comprehensive recording of clinical endpoints.

Our study has some limitations, however. First, not all participants in the trial had follow-up HBV viral loads. Viral load was only measured in HBsAg positive participants receiving dexamethasone, or in HBsAg negative/HBcAb positive participants (receiving blinded study drug). HBV load was not routinely measured in HBsAg positive placebo-treated participants. Those who died early from tuberculous meningitis also missed testing. Consequently, more severely unwell participants may have been excluded from this evaluation of follow-up viral load and reactivation.

Second, the follow-up testing time point was not consistent for all participants. For the “3-month” testing time point, a pragmatic window of 14–104 days was used (defined as “2 weeks, to 3 months plus 2 weeks”). HBV load may have been elevated at time points where viral load measurements were not performed, including early in anti-TB chemotherapy where the highest doses of dexamethasone are given. However, given this was not reflected in peak ALT or bilirubin values, nor in clinical DILI events, this seems unlikely. Additionally, dexamethasone may be utilized many months into anti-TB treatment for neuroinflammatory complications, and viral load elevation may have developed after 3 months. However, 12-month endpoint data do not support this suggestion.

Third, monitoring of ALT, bilirubin, and clinical liver injury events may be confounded by antituberculosis drugs (rifampicin, isoniazid, pyrazinamide), which can all elevate LFTs and cause liver injury. Finally, we were unable to differentiate active from past hepatitis C infection in individuals with positive antibodies, nor evaluate the impact of corticosteroids on hepatitis C. However, positive hepatitis C antibodies were uncommon in our analysis groups, and none were seen in individuals who were HBsAg positive.

In conclusion, adjunctive dexamethasone for tuberculous meningitis did not increase the risk of laboratory or clinical HBV reactivation, nor any downstream clinical adverse events. Use of tenofovir alongside dexamethasone for individuals with HBsAg seropositivity was safe, and may be an appropriate strategy in HBV chronic infection when corticosteroids are deemed necessary.

## Supplementary Material

ofag575_Supplementary_Data

## Supplementary Data

Supplementary materials are available at *Open Forum Infectious Diseases* online. Consisting of data provided by the authors to benefit the reader, the posted materials are not copyedited and are the sole responsibility of the authors, so questions or comments should be addressed to the corresponding author.
