# Supplementary material for: Risk of Hepatitis B Reactivation Following Corticosteroids for Tuberculous Meningitis
Source: Open Forum Infect Dis. 2026 Sep 2;13(9):ofag575. doi: 10.1093/ofid/ofag575 (PMC13579466; doi:10.1093/ofid/ofag575)
Supplement: Supplementary file 1 [file ofag575_supplementary_data.docx]

**Supplementary figure 1: Unblinding and tenofovir schema**

|  | **TT-genotype** | **CT-genotype** | **CC-genotype** |
| --- | --- | --- | --- |
| **HBsAg +ve** | Participant will receive open label dexamethasone   - Participant will also receive **tenofovir.** | Randomise then UNBLIND   - If study drug is DEXAMETHASONE participant will also receive **tenofovir.** - If study drug is PLACEBO, participant will **NOT** receive tenofovir (unless otherwise clinically indicated). | Randomise then UNBLIND   - If study drug is DEXAMETHASONE participant will also receive **tenofovir.** - If study drug is PLACEBO, participant will **NOT** receive tenofovir (unless otherwise clinically indicated). |
| **HBsAg –ve,**  **anti-HBc +ve** | Participant will receive open label dexamethasone   - Tenofovir treatment is at the discretion of the treating clinician. | Randomise  Do NOT unblind   - In the unlikely event that hepatitis B viral load testing shows detectable hepatitis B virus then a discussion should take place about unblinding, and whether tenofovir would be given to the participant if the study drug was shown to be DEXAMETHASONE. | Randomise  Do NOT unblind   - In the unlikely event that hepatitis B viral load testing shows detectable hepatitis B virus then a discussion should take place about unblinding, and whether tenofovir would be given to the participant if the study drug was shown to be DEXAMETHASONE. |
| **HBsAg –ve,**  **anti-HBc -ve** | Participant will receive open label dexamethasone   - Participant will **NOT** receive tenofovir**.** | Randomise  Do NOT unblind   - Participant will **NOT** receive tenofovir**.** | Randomise  Do NOT unblind   - Participant will **NOT** receive tenofovir**.** |
